# PAN-811 Blocks Chemotherapy Drug-Induced *In Vitro* Neurotoxicity, While Not Affecting Suppression of Cancer Cell Growth

**DOI:** 10.1155/2016/9392404

**Published:** 2015-11-10

**Authors:** Zhi-Gang Jiang, Steven A. Fuller, Hossein A. Ghanbari

**Affiliations:** Panacea Pharmaceuticals, Inc., 209 Perry Parkway, Suite 13, Gaithersburg, MD 20877, USA

## Abstract

Chemotherapy often results in cognitive impairment, and no neuroprotective drug is now available. This study aimed to understand underlying neurotoxicological mechanisms of anticancer drugs and to evaluate neuroprotective effects of PAN-811. Primary neurons in different concentrations of antioxidants (AOs) were insulted for 3 days with methotrexate (MTX), 5-fluorouracil (5-FU), or cisplatin (CDDP) in the absence or presence of PAN-811·Cl·H_2_O. The effect of PAN-811 on the anticancer activity of tested drugs was also examined using mouse and human cancer cells (BNLT3 and H460) to assess any negative interference. Cell membrane integrity, survival, and death and intramitochondrial reactive oxygen species (ROS) were measured. All tested anticancer drugs elicited neurotoxicity only under low levels of AO and elicited a ROS increase. These results suggested that ROS mediates neurotoxicity of tested anticancer drugs. PAN-811 dose-dependently suppressed increased ROS and blocked the neurotoxicity when neurons were insulted with a tested anticancer drug. PAN-811 did not interfere with anticancer activity of anticancer drugs against BNLT3 cells. PAN-811 did not inhibit MTX-induced death of H460 cells but, interestingly, demonstrated a synergistic effect with 5-FU or CDDP in reducing cancer cell viability. Thus, PAN-811 can be a potent drug candidate for chemotherapy-induced cognitive impairment.

## 1. Introduction

One of the most common complications of chemotherapeutic drugs is toxicity to the central nervous system (CNS), namely, chemotherapy-induced cognitive impairment or chemobrain. This toxicity can present in many ways, including encephalopathy syndromes and confusional states, seizure activity, headache, cerebrovascular complications and stroke, visual and hearing loss, cerebellar dysfunction, and spinal cord damage with myelopathy [1]. Mild to moderate effects of chemotherapy on cognitive performance occur in 15–50% of the survivors after treatment [2, 3]. The cognitive problems can last for many years after the completion of chemotherapy in a subset of cancer survivors. Up to 70% of patients with cancer report that these cognitive difficulties persist well beyond the duration of treatment [4–6]. Chemobrain can seriously affect quality of life and life itself in cancer patients.

Among possible candidate mechanisms, oxidative stress (OS) may play a key role in cognitive disorders caused by broad types of anticancer drugs, such as antimetabolites, mitotic inhibitors, topoisomerase inhibitors, and paclitaxel [7]. These chemotherapeutic agents are not known to rely on oxidative mechanisms for their anticancer effects. Among the antimetabolite drugs, methotrexate (MTX) and 5-fluorouracil (5-FU), widely used chemotherapeutic agents, are most likely to cause CNS toxicity [1]. Although there are yet no reports of 5-FU increasing CNS OS, it has been observed to induce apoptosis in rat cardiocytes through intracellular OS [8], to increase OS in the plasma of liver cancer patients [9], and to decrease glutathione (GSH) in bone marrow cells [10]. MTX can also cross the blood-brain barrier as well [11] and result in an increase of OS in cerebral spinal fluid and executive dysfunction in MTX-treated patients of pediatric acute lymphoblastic leukemia [12, 13]. It is well known that ROS, such as H_2_O_2_, can result in neuronal cell death [14, 15]. Cisplatin (CDDP) is an alkylating agent. Its cytotoxic effect is thought to be mediated primarily by the generation of nuclear DNA adducts, which, if not repaired, cause cell death as a consequence of DNA replication and transcription blockage. However, oxidative damage has been observed* in vivo* following exposure to CDDP in several tissues including nervous tissue, suggesting a role for OS in the pathogenesis of CDDP-induced dose-limiting toxicities [16–18]. Cotreatment with antioxidants (AOs) suppresses the toxic effects of CDDP on several organs [19, 20].

Currently, there are no proven treatments for chemotherapy-induced cognitive impairment. Some efforts have been focused on correcting cognitive deficits rather blocking the neurotoxic pathway of chemotherapeutic drugs [21]. Since ROS mediates neurotoxicity in a number of neurodegenerative disorders, one strategy in disease control has been focused on development of antioxidants as preventive and therapeutic molecules. These include vitamin C, vitamin E, glutathione, coenzyme Q (CoQ), carotenoids, melatonin, and green tea extract [22, 23]. In contrast to the minimal positive effects of these efforts, antioxidative therapy could be a promising strategy for the treatment of neurotoxicity. Several preclinical studies have shown that AO treatment prevents chemotherapy-induced OS and cognitive deficits when administered prior to and during chemotherapy [24, 25].

Our previous research has demonstrated that PAN-811 (known as 3-aminopyridine-2-carboxaldehyde thiosemicarbazone or Triapine), a bioavailable small molecule (MW 195) currently in phase II clinical trials for the treatment of patients with cancer, can efficiently block neurodegeneration. Major underlying mechanisms for the neuroprotection of PAN-811 are blockage of both excitatory pathway and OS [22]. Hence we hypothesized that PAN-811 could protect neurons from anticancer drugs, such as MTX, 5-FU, and CDDP. Since PAN-811 is an anticancer drug targeting ribonucleotide reductase, which is distinctive from intracellular targets of MTX, 5-FU, or CDDP, coadministration of PAN-811 with any of these may also have a synergistic effect on suppression of cancer cell growth.

## 2. Materials and Methods

### 2.1. Neuronal Cell Culture

Mixed cortical and striatal neurons from embryonic day 17 male Sprague-Dawley rats (tissue obtained from NIH) were seeded into poly-D-lysine coated 96-well plates at density of 50,000 cells/well and initially cultured at 37°C, 5% CO_2_, in neurobasal medium (NB) with B27 supplement (Invitrogen) containing full strength of AOs to obtain highly enriched (95%) neurons [26]. Since AOs, including vitamin E, vitamin E acetate, superoxide dismutase (SOD), catalase (CAT), and GSH, are additives to culture medium, reduction of AO concentration in culture medium provides an approach to determine the level of OS involvement in a neurotoxic process. In our study, the culture medium was replaced at a 50% ratio with NB plus B27 minus AOs twice at days 7 and 9 to set AO concentrations as 50% and 25%, respectively. At 16 days* in vitro* (d.i.v.), a fraction of the culture medium was harvested for lactate dehydrogenase (LDH) assay, and then AO concentration was reduced to 12.5% or 17.5%, and cultured for a further 5 hours prior to ending the experiment.

### 2.2. Cancer Cell Culture

The mouse liver cancer cell line BNLT3 (gift of Dr. Jack Wands, Brown University) and the human lung cancer cell line H460 (ATCC) were seeded into 96-well plates at a density of 4,000 cells/well and cultured at 37°C, 5% CO_2_, in DMEM (11965, Gibco) supplemented with 10% fetal bovine serum, 20 mM HEPES, 1 mM sodium pyruvate, and 24 ng/mL gentamycin (all reagents came from Gibco).

### 2.3. Cell Insults and Treatments

Determination of concentration for each anticancer drug in our experiments was based on its reported concentration in human cerebral spinal fluid (CSF) in chemotherapy, literature report of its neurotoxicity in culture, and our preliminary* in vitro* experimental data. At 13 d.i.v., the neuronal cell cultures were insulted with 100 *μ*M of MTX (M9929, Sigma) [27–30], 25 *μ*M of 5-FU (F6627, Sigma) [31–33], or 3.5 *μ*M of CDDP (sc-200896, Santa Cruz) [34, 35] for 3 days in absence or presence of PAN-811·Cl·H_2_O. For ROS examination, the neurons were insulted with both 100 *μ*M of MTX and 25 *μ*M of 5-FU by 15 d.i.v. PAN-811·Cl·H_2_O was added to cultures to final concentrations of 1.25, 2.5, 5, and 10 *μ*M at the same time as addition of the anticancer drugs. For cancer cell lines, the cells were insulted by the second day of cell seeding with the same concentration of MTX, 5-FU, or CDDP as used in neuronal culture in the absence or presence of 10 *μ*M PAN-811·Cl·H_2_O for another 3 days.

### 2.4. Quantitative Assays and Morphological Assessment

Cell membrane integrity and mitochondrial function of either neurons or cancer cells were measured with LDH and 3-(4,5-dimethylthiazol-2-yl)-5-(3-carboxymethoxyphenyl)-2-(4-sulfophenyl)-2H-tetrazolium [MTS] analyses, respectively. The latter has been used to quantify cell survival. For the LDH assay, a mixture of a 35 *μ*L aliquot of culture supernatant and 17.5 *μ*L of Mixed Substrate, Enzyme and Dye Solutions (Sigma) was incubated at room temperature (RT) for 30 minutes. For the MTS assay, 10 *μ*L of MTS reagent (Promega) was added to a culture well containing neurons in 50 *μ*L of medium. The preparations were incubated at 37°C for 2 hours. The preparations for both assays were then spectrophotometrically measured at 490 nm using a 96-well plate reader (Mode 550, Bio-Rad). Neuronal cell death was morphologically determined based on the integrity of the cell soma and continuity of neuronal processes. The change in number of cancer cells was judged directly by cell density. Cells were photographed under an inverted phase contrast microscope (IX 70, Olympus) using 10x or 20x objective.

### 2.5. ROS Examination

Neurons were incubated in 15 *μ*M dihydrorhodamine 123 (DHR123, Molecular Probes) for 30 min at 37°C to determine intramitochondrial ROS levels. Fluorescence was photographed by using a fluorescent microscope and quantified by excitation at 485 nm and emission at 520 nm using a 96-well plate reader (Model 550, Bio-Rad).

### 2.6. Data Analysis

Data were generated from 4–6 replicate wells, expressed as mean ± standard deviation (SD), and statistically evaluated at a significance level of 1% with one-factor ANOVA or Student's *t*-test by using software VASSARSTATS (http://vassarstats.net/) followed by the Tukey HSD test. Figure symbols are as follows: #, *P* < 0.05, and ##, *P* < 0.01, compared with control; *∗*, *P* < 0.05, and *∗∗*, *P* < 0.01, compared with the insulted group; §§, *P* < 0.01, compared with PAN-811 treated group by Student's *t*-test.

## 3. Results

### 3.1. MTX, 5-FU, or CDDP Elicited Neurotoxicity in an AOs-Dependent Manner

**Figure 1:**
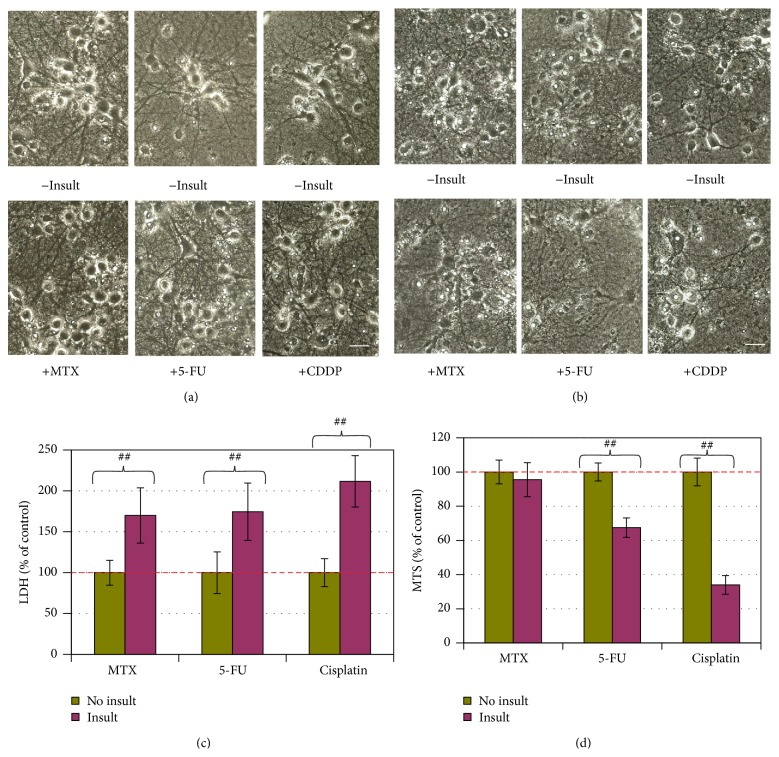
Neurotoxicity of MTX, 5-FU, or CDDP in an AO-dependent manner. (a, b) Phase contrast photographs for neurons in 25% AO and 12.5% AO, respectively (bar = 25 *μ*m). (c) Cell membrane leakage was determined via the LDH analysis at the end of experiment for neurons in 25% AO (*n* = 5). (d) Cell viability was determined with MTS analysis for neurons in 12.5% AO (*n* = 5). The bar in green and bars in other colors indicate the cultures without an insult and with anticancer drug insults, respectively. LDH and MTS data are expressed as % of noninsulted control. Figure symbol is ##,  *P* < 0.01, compared with noninsult control group by Student's *t*-test.

By 3 days following the insults, neither MTX at 100 *μ*M, 5-FU at 25 *μ*M, nor CDDP at 3.5 *μ*M caused morphological changes, LDH release, or MTS reduction when neurons were cultured in the medium containing 100% or 50% AOs (data not shown). However, MTX, 5-FU, or CDDP at the same concentrations elicited significant LDH increase (indicating cell membrane leakage, Figure 1(c)) in the culture supernatant when the AO concentration was reduced to 25%, although no cell damage was visible (Figure 1(a)), and no change in MTS level was detectable (data not shown) under these conditions. When the AO concentration was reduced to 12.5% for 5 hours at 16 d.i.v., extensive neuronal cell death occurred in the cultures insulted with 25 *μ*M 5-FU or 3.5 *μ*M CDDP, as indicated by loss of cell bodies, together with interruption of neurite networks on the background (Figure 1(b)). Corresponding to the morphological cell death, the MTS readings for 5-FU- and CDDP-insulted groups were reduced by 27% and 66%, respectively (Figure 1(d)). MTX at 100 *μ*M did not elicit significant MTS reduction (Figure 1(d)) under 12.5% AO condition. Thus, the neurotoxicities elicited with MTX, 5-FU, or CDDP were dependent on AO reduction.

### 3.2. PAN-811 Dose-Dependently Suppresses MTX-, 5-FU-, or CDDP-Induced Neurotoxicity

**Figure 2:**
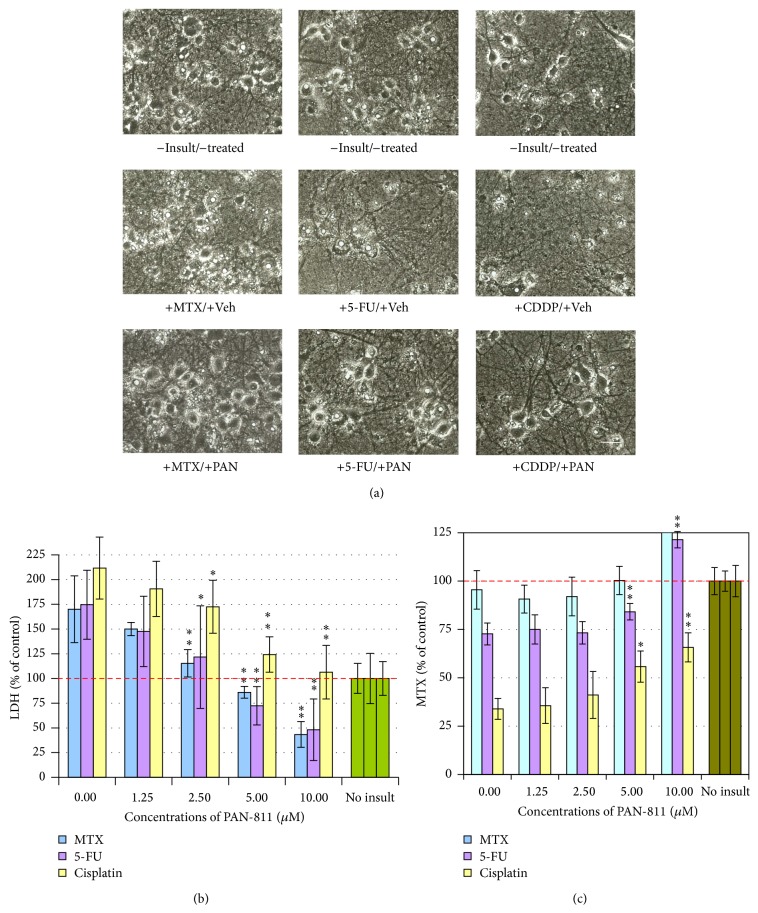
Dose-dependent neuroprotection of PAN-811·Cl·H_2_O against anticancer drug-induced neurotoxicity. (a) Phase contrast photographs for neurons in 12.5% AO (bar = 25 *μ*m; PAN: PAN-811·Cl·H_2_O). (b) LDH analysis for (a) (*n* = 5). (c) MTS analysis for (a) (*n* = 6). The bar in green and bars in other colors in the graphs indicate the cultures without an insult and with anticancer drug insults, respectively. Data are expressed as % of noninsulted control. Figure symbols are *∗*, *P* < 0.05, and *∗∗*, *P* < 0.01, compared with insult group alone (without PAN-811 treatment) by one-factor ANOVA followed with Tukey HSD test.

We then examined PAN-811 for its effect on the anticancer drug-induced neurotoxicity at the 12.5% AO condition. MTX at 100 *μ*M did not result in significant loss of cell number, while 5-FU at 25 *μ*M or CDDP at 3.5 *μ*M caused robust loss of neurons in culture (Figure 2(a)). Correspondingly, MTX insult did not significantly affect the MTS reading, while 5-FU- and CDDP-insulted cultures showed significant reduction in MTS readings (Figure 2(c)). PAN-811 dose-dependently inhibited 5-FU- or CDDP-induced MTS reduction. PAN-811 at 10 *μ*M completely blocked 5-FU-induced MTS reduction and inhibited CDDP-induced MTS reduction by 48%. The LDH release assay demonstrated that each of MTX at 100 *μ*M, 5-FU at 25 *μ*M, and CDDP at 3.5 *μ*M resulted in significant increases in LDH reading (Figure 2(b)). PAN-811 dose-dependently suppressed LDH increase caused by each anticancer drug. PAN-811 at 5 *μ*M fully blocked LDH release in MTX-, 5-FU-, or CDDP-insulted cultures (with no statistically significant difference from untreated control culture by ANOVA analysis). Thus, PAN-811 was demonstrated as a potential neuroprotective compound for anticancer drug MTX-, 5-FU-, or CDDP-induced neurotoxicity.

### 3.3. PAN-811 Suppresses Cell Membrane Leakage When MTX and 5-FU Are Coadministered

**Figure 3:**
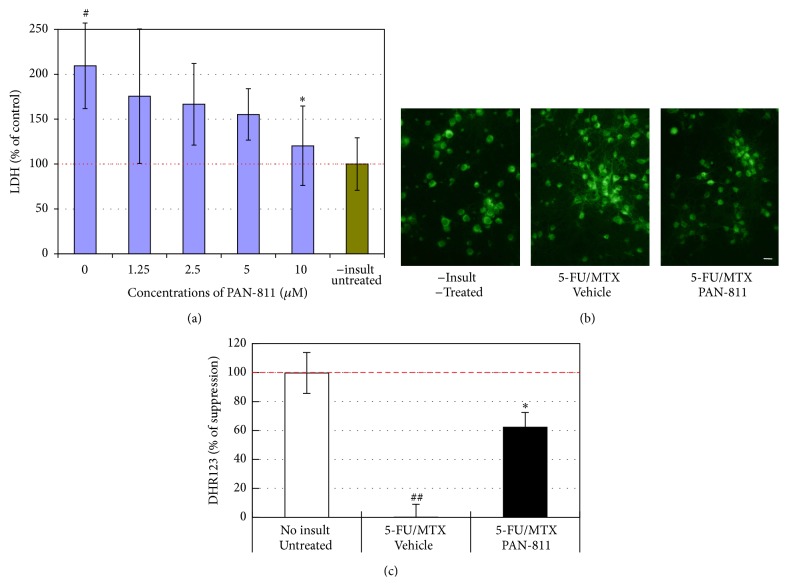
Suppression of 5-FU/MTX-induced increases in LDH and DHR123 readings by PAN-811. (a) LDH release analysis for neurons that were cultured in 17.5% AOs-containing medium and insulted with both 100 *μ*M MTX and 25 *μ*M 5-FU in the absence or presence of PAN-811·Cl·H_2_O at different concentrations for 1 day (blue bars *n* = 6); Green bar represents noninsult/untreated control. (b) Fluorescent microscope for neurons in 17.5% AOs-containing medium insulted with both 100 *μ*M MTX and 25 *μ*M 5-FU in the absence or presence of 10 *μ*M PAN-811·Cl·H_2_O for 1 day and incubated with DHR123 for 30 min (bar = 50 *μ*m). (c) Quantification of (b) at excitation at 485 nm and emission at 520 nm (*n* = 4). Data are expressed as % suppression = [(Insulted&Untreated − Insulted&Treated)/(Insulted&Untreated − NonInsulted&Untreated) *∗* 100%]. Figure symbols are *∗*, *P* < 0.05, compared with insult group alone (without PAN-811 treatment) by Student's *t*-test (one tail) and one-factor ANOVA followed by Tukey HSD test; #, *P* < 0.05; ##, *P* < 0.01, compared with noninsult/untreated control group by one-factor ANOVA followed with Tukey HSD test.

Since MTX and 5-FU are coadministered for cancer therapies in many cases, we were interested to know if PAN-811 can block neurotoxicity that is elicited with a combined insult with both MTX and 5-FU. An insult with a combined 100 *μ*M MTX and 25 *μ*M 5-FU resulted in a 109% increase in LDH reading by comparison with noninsulted control group (*P* < 0.05 by ANOVA; Figure 3(a)). PAN-811 showed concentration-dependent suppression of LDH release within the tested range from 1.25 to 10 *μ*M. PAN-811 at 10 *μ*M fully inhibited MTX/5-FU-elicited LDH increase.

### 3.4. PAN-811 Inhibits MTX- and 5-FU-Elicited OS

To understand the underlying mechanism for MTX- and 5-FU-induced neurotoxicity, a cell-permeable fluorogenic probe DHR123 was used for the detection of intramitochondrial ROS. Neuronal insult with coadministered 100 *μ*M MTX and 25 *μ*M 5-FU greatly increased intensity of DHR123 fluorescence (Figure 3(b)), resulting in a 33.4% increase in DHR123 level in comparison with noninsulted group (*P* < 0.05 by *t*-test, data not shown). PAN-811 at 10 *μ*M provided significant suppression to the increased ROS, showing a 62.3% suppression rate (Figure 3(c)).

### 3.5. PAN-811 Shows No Antagonistic Effect on MTX-, 5-FU-, or CDDP-Induced Cytotoxicity in BNLT3 Cells

To understand whether PAN-811 could interfere with anticancer efficacy of tested anticancer drugs, the mouse liver cancer cell line BNLT3 was cotreated with each anticancer drug at the concentrations used for elicitation of neurotoxicity and 10 *μ*M PAN-811, the highest concentration used for neuronal protection in these experiments.

**Figure 4:**
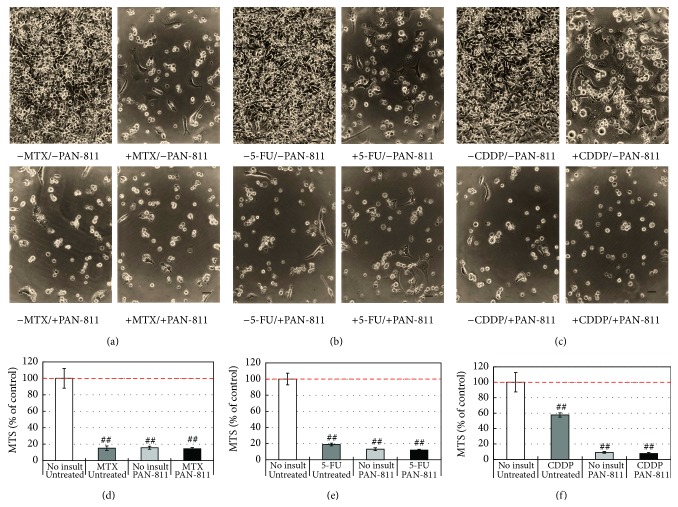
No interference of PAN-811·Cl·H_2_O with anticancer drug-induced cytotoxicity to mouse cancer cell line BNLT3. (a)–(c) Phase contrast photographs for BNLT3 cells that were treated without or with 10 *μ*M PAN-811·Cl·H_2_O and insulted with 100 *μ*M MTX, 25 *μ*M 5-FU, or 3.5 *μ*M CDDP for 3 days, respectively (bar = 50 *μ*m). (d)–(f) MTS analysis corresponding to (a)–(c) (*n* = 6). Data are expressed as % of noninsulted/untreated control. Figure symbol is ##, *P* < 0.01, compared with noninsult/untreated control group by one-factor ANOVA followed with Tukey HSD test.

A 3-day insult with 100 *μ*M MTX severely reduced the cancer cell number (Figure 4(a)). In the culture treated with 10 *μ*M PAN-811 alone or cotreated with 100 *μ*M MTX and 10 *μ*M PAN-811, cell density was also much lower than that in no-insult control. Quantitatively, MTX at 100 *μ*M reduced MTS reading by 85% (Figure 4(d)), while PAN-811 at 10 *μ*M reduced MTS reading to the same level as MTX. A cotreatment with both did not cause any further reduction in MTS reading when comparing with MTX alone.

Similarly, 5-FU at 25 *μ*M significantly reduced the cell density of the cancer cells, and a cotreatment with both 25 *μ*M 5-FU and 10 *μ*M PAN-811 significantly decreased the cell number as well (Figure 4(b)). Quantitatively, 5-FU at 25 *μ*M reduced MTS reading by 84%, which was less efficient than 10 *μ*M PAN-811 group (Figure 4(e)). A cotreatment with both caused a further reduction in MTS reading when comparing with 5-FU alone. No synergistic effect between 5-FU and PAN-811 could be detected.

An insult with 3.5 *μ*M CDDP also caused a decrease in the cell density (Figure 4(c)), while a treatment with PAN-811 alone or a cotreatment with both 3.5 *μ*M CDDP and 10 *μ*M PAN-811 introduced a significant reduction in the cell density. Quantitatively, 3.5 *μ*M CDDP reduced MTS reading by 44%, while 10 *μ*M PAN-811 caused a 94% reduction in MTS reading (Figure 4(f)). A cotreatment with both did not introduce an extra reduction in MTS reading by comparing with PAN-811 alone, despite showing much lower reading than CDDP alone (*P* < 0.01).

In general, PAN-811 did not show any inhibition in the effect of MTX, 5-FU, or CDDP on BNLT3 cells, neither did it demonstrate any synergistic effect with each tested anticancer drug on BNLT3 cell growth.

### 3.6. PAN-811 Shows No Antagonistic Effect on MTX-, 5-FU-, or CDDP-Induced Cell Death of H460 Cells, While Demonstrating a Synergistic Effect with 5-FU or CDDP on Suppression of the Cell Growth

To understand whether there is any negative effect of PAN-811 on the efficacy of tested anticancer drugs in humans, the human lung cancer cell line H460 was treated with each of these anticancer drugs at the concentrations used for elicitation of neurotoxicity, in the absence or presence of 10 *μ*M PAN-811.

**Figure 5:**
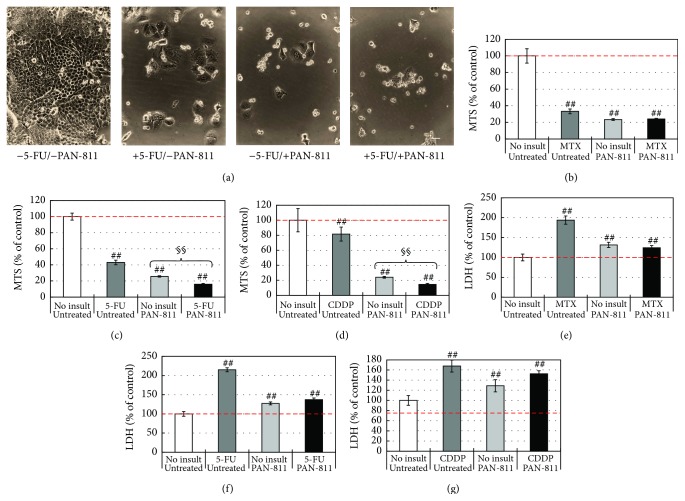
Effects of PAN-811·Cl·H_2_O on anticancer drug-induced cytotoxicities to human cancer cell H460. (a) Phase contrast photographs for the H460 cells that received 25 *μ*M 5-FU, 10 *μ*M PAN-811, or both for 3 days (bar = 50 *μ*m). (b)–(d) MTS analysis for the H460 cells that received 10 *μ*M PAN-811·Cl·H_2_O, one of 100 *μ*M MTX, 25 *μ*M 5-FU, and 3.5 *μ*M CDDP, or both 10 *μ*M PAN-811·Cl·H_2_O and one of these anticancer drugs for 3 days, respectively (*n* = 6). (e)–(g) LDH analysis for (b)–(d) (*n* = 6). Data are expressed as % of noninsulted/untreated control. Figure symbol is ##, *P* < 0.01, compared with noninsult/untreated control group by one-factor ANOVA followed with Tukey HSD test. §§, *P* < 0.01 between no insult and insult groups given PAN-811 by Student's* t*-test.

A 3-day insult with 100 *μ*M MTX, 10 *μ*M PAN-811, or both robustly decreased the cell density of H460 in culture (data not shown). Quantitatively, 100 *μ*M MTX and 10 *μ*M PAN-811 reduced MTS readings by 67% and 76%, respectively. The MTS reading for a cotreatment with both 100 *μ*M MTX and 10 *μ*M PAN-811 was about the same as 10 *μ*M PAN-811 alone (Figure 5(b)). In membrane integrity analysis (Figure 5(e)), 100 *μ*M MTX resulted in a 95% increase in LDH reading in the culture supernatant, while 10 *μ*M PAN-811 led to a 31% increase in the LDH reading. A cotreatment with 100 *μ*M MTX and 10 *μ*M PAN-811 reduced LDH reading by 70% when compared with MTX group (*P* < 0.01 by ANOVA), indicating an inhibitory effect of PAN-811 on MTX-caused membrane leakage.

Similarly, a 3-day treatment with 25 *μ*M 5-FU, 10 *μ*M PAN-811, or both robustly decreased the cell density of H460 in culture (Figure 5(a)). Quantitatively, 25 *μ*M 5-FU and 10 *μ*M PAN-811 reduced MTS readings by 57% and 74%, respectively (Figure 5(c)). In contrast, a cotreatment with 25 *μ*M 5-FU and 10 *μ*M PAN-811 reduced MTS readings by 84%, which shows a statistically significant difference from 5-FU (*P* < 0.01) or PAN-811 alone (*P* < 0.01), indicating a synergistic effect of 5-FU and PAN-811 on suppression of growth of human lung cancer cell H460. In membrane integrity analysis (Figure 5(f)), 25 *μ*M 5-FU resulted in a 124% increase in LDH reading in the culture supernatant, while 10 *μ*M PAN-811 led to a 30% increase in the LDH reading. A cotreatment with 100 *μ*M 5-FU and 10 *μ*M PAN-811 enhanced LDH reading by 40%, which is much lower than that in the group with 25 *μ*M 5-FU alone. It indicates an inhibitory effect of PAN-811 on 5-FU-caused membrane leakage.

A 3-day treatment with 3.5 *μ*M CDDP, 10 *μ*M PAN-811, or both greatly decreased the cell density of H460 in culture (data not shown). Quantitatively, 3.5 *μ*M CDDP and 10 *μ*M PAN-811 reduced MTS readings by 22% and 75%, respectively (Figure 5(d)). A cotreatment with 3.5 *μ*M CDDP and 10 *μ*M PAN-811 reduced MTS readings by 85%, which shows a statistically significant difference from CDDP (*P* < 0.01 by ANOVA) or PAN-811 alone (*P* < 0.01 by *t*-test), indicating a synergistic effect of CDDP and PAN-811 on suppression of growth of human lung cancer cell H460. In membrane integrity analysis (Figure 5(g)), 3.5 *μ*M CDDP resulted in a 71% increase in LDH reading in the culture supernatant, while 10 *μ*M PAN-811 led to a 30% increase in the LDH reading. A cotreatment with 3.5 *μ*M CDDP and 10 *μ*M PAN-811 enhanced LDH reading by 57%, which is a statistically significant difference from that in the group with 3.5 *μ*M CDDP alone (*P* < 0.01), demonstrating an inhibitory effect of PAN-811 on CDDP-induced membrane leakage.

In general, PAN-811 did not show any inhibition in the effect of MTX, 5-FU, or CDDP on cell growth of H460 cells, although it manifested an inhibitory effect on MTX-, 5-FU- or CDDP-induced membrane leakage. A synergistic effect between 5-FU and PAN-811 or between CDDP and PAN-811 occurred on suppression of H460 cell survival.

## 4. Discussion

The fate of neurons under an OS condition is dependent on the balance between production of ROS and strength of AO defense systems* in vivo*. Enzymatic AOs, such as SOD and CAT, and nonenzymatic AOs, exemplified with vitamin E and GSH, are both involved in the defenses [23]. Loss of the balance, under condition such as chemotherapy, can elicit cytotoxicity and organ toxicity in experimental animals [24, 36] and in humans [9, 12, 13]. Administration of anticancer drugs is accompanied by not only an increase in ROS level, but also a decrease in antioxidative enzymes [37, 38]. In comparison with the* in vivo *studies, it is rare to find an* in vitro* study that examines the direct effects of anticancer drug on neurons in an enriched neuronal culture system. The presence of AOs in the culture medium may sufficiently block the effect of an anticancer drug and therefore the system is not suitable for examining ROS-mediated neurotoxicity and may not reflect the real conditions under chemotherapy in animals and humans. To mimic* in vivo* conditions under chemotherapy, we reduced AO concentrations in a double-diluted manner to a final AO concentration of 12.5%. It was observed that neurotoxicity of MTX, 5-FU, or CDDP occurred only when neurons were bathed in low AO-containing medium. Cell membranes seemed to be more fragile to these anticancer drugs under these conditions. When the AO content was reduced to 25%, MTX, 5-FU, and CDDP all resulted in robust LDH release, but neither notable morphological changes nor MTS reading differences in anticancer drug-insulted groups were detected. Only when the AO content was further reduced to 12.5% was there observation of morphological cell death in 5-FU- or CDDP-insulted groups and corresponding 33% and 66% reductions in MTS readings, respectively. These data, together with the phenomenon where coadministration of MTX and 5-FU resulted in a significant intramitochondrial ROS increase, indicate a key role of OS in mediation of the* in vitro* neurotoxicity.

Our study demonstrated that under low AO conditions MTX insult only resulted in membrane leakage but did not show significant detrimental effects on cell viability of neurons in our neuron-enriched culture. This is identical to the previous findings that excitatory neurotoxicity marks MTX-mediated cell death, which only occurs in the presence of glial cells, and is protected by N-methyl-D-aspartate receptor antagonists MK-801 and memantine [39].

PAN-811 can suppress neurotoxicity of all tested anticancer drugs in the present study. Under 12.5% AO condition, PAN-811 dose-dependently blocked MTX-, 5-FU-, or CDDP-induced membrane leakage. In addition, PAN-811 at 10 *μ*M fully inhibited 5-FU-induced MTS reduction and elevated MTS reading by 48% for CDDP-insulted neurons under 12.5% AO condition. Furthermore, neurons that were treated with PAN-811 looked to have a healthy appearance even when they were insulted with MTX, 5-FU, or CDDP. Our previous studies have demonstrated that, besides inhibiting excitatory neurotoxicity, PAN-811 can protect neurons from cell death under different OS-involved conditions, such as hypoxia [22], and hydrogen peroxide insult [14, 15]. In a cell-free and metal-free system, PAN-811 demonstrated an activity in direct scavenging of stable radical diphenylpicrylhydrazyl (DPPH) [22]. Taken together, the neuroprotection provided by PAN-811 in the anticancer drug-insulted condition is most likely due to its activity in inhibition of intracellular ROS accumulation.

In this study, the blockage of oxidative damage by PAN-811 was shown by not only its neuroprotective effect but also its inhibitory role in anticancer drug-induced membrane leakage. Our results showed that each of MTX, 5-FU, and CDDP can induce membrane leakage of cancer cell H460. Theoretically, ROS can be produced in plasma membrane and other cell compartments [40]. Free radicals can pass freely through cellular and nuclear membranes and oxidize biomacromolecules, including lipids. Lipid peroxidation caused by ROS leads to membrane leakage [41]. Efficient inhibition of membrane leakage of H460 cells by PAN-811 indicates its role in suppression of ROS signal not only in neurons but also in other cell types.

Our result demonstrated that PAN-811 did not suppress anticancer efficacy of anticancer drugs MTX, 5-FU, and CDDP despite suppressing anticancer drug-induced membrane leakage. This indicates that the anticancer activity of the tested anticancer drugs does not rely on intramitochondrial ROS accumulation they induced, which provides a basis for using PAN-811 as a neuroprotectant in chemotherapy. In addition, PAN-811 manifested a synergistic effect with 5-FU or CDDP on suppression of cancer cell growth. Both MTX and 5-FU are antimetabolites or antifolate drugs. MTX inhibits DNA synthesis by competitively binding to dihydrofolate reductase, an enzyme that converts dihydrofolate into tetrahydrofolate [42]. 5-FU acts predominantly as a thymidylate synthase inhibitor and suppresses synthesis of the pyrimidine thymidine, which is a nucleoside required for DNA replication [43]. CDDP is an alkylating agent. It binds to and causes cross-linking of DNA, which ultimately triggers apoptosis [44]. PAN-811 is an anticancer drug by itself with a different intracellular target from MTX, 5-FU, and CDDP. PAN-811 divalently chelates the ferrous ions of ribonucleotide reductase and blocks its bioactivity in conversion of ribonucleotides to deoxynucleotides and therefore inhibits DNA synthesis [45]. The synergistic effect by cotreatment with PAN-811 and 5-FU or CDDP may be due to affecting more than one intracellular target. The synergistic effect by cotreatment with PAN-811 may provide an opportunity in reduction of dose usage of 5-FU or CDDP. In this way, the neurotoxicity of 5-FU or CDDP could be further reduced while retaining equal strength of anticancer efficacy. PAN-811 can be a potential neuroprotective drug for chemotherapy-induced cognitive impairment due to its* in vitro* inhibition of MTX-, 5-FU-, or CDDP-induced neurotoxicity. A pharmacodynamic study for the effect of PAN-811 on cognitive functions will be carried out in a chemobrain animal model in near future.
